# Examination of customized questioned digital documents

**DOI:** 10.1111/1556-4029.15703

**Published:** 2025-02-07

**Authors:** Oluwasola Mary Adedayo, Martin S. Olivier

**Affiliations:** ^1^ Department of Applied Computer Science The University of Winnipeg Winnipeg Canada; ^2^ Department of Computer Science University of Pretoria Hatfield Pretoria South Africa

**Keywords:** alternative approach to digital forensics, database‐customized documents, digital aspects of questioned document examination, digital document exemplars, digital forensics, document analysis, document recreation, forensic questioned digital document, questioned digital document examination, questioned document examination

## Abstract

With the increasing trend of digitization of business processes and personal communication across the globe, digital documents of intrinsic value continue to be created. Whereas the questioned document examination (QDE) field of forensic science deals with the examination of “physical” documents potentially disputed in a court of law, there are no developed approaches for handling questioned digital documents (QDDs). Although techniques that address related problems such as identifying document types and image forensics exist, concrete strategies for analyzing questioned “digital” documents still need to be developed. This paper focuses on developing methods to examine QDDs that are customized from a database, due to the versatile use of customized documents in many areas. As a basis for our approach, we make the case for the need to develop analysis techniques for a digital counterpart of QDE which we term Questioned Digital Document Examination (QDDE). We posit that there is a benefit in considering digital aspects of forensic science disciplines where the questions answered by the discipline are clear, from a digital perspective. The paper describes some of the aspects that can be considered in the domain of question digital document examination. In designing methods for QDDE, we discuss the process of document recreation and describe the feasibility of our recreation process in different scenarios. Our experiments show that an alternative approach of considering digital aspects from a well‐defined physical domain is worthwhile. It also supports the practical application of our approach in examining documents customized from a database.

**Highlights**

- An alternative approach for narrowing the questions in digital forensic examinations.
- Digital aspects of questioned document examination (QDE).
- Integrating physical and digital forensics approaches in forensic investigations.
- Applying database reconstruction methods and revision identifiers to questioned digital document.
- Formal process and document recreation for database‐customized digital document examination.

## 1 INTRODUCTION

In many forensic science disciplines, there is a clear understanding of the type of legal questions that can be answered or problems that can be addressed by the discipline. Fundamental concepts and principles that govern the field and apply to forensic analysis in different disciplines are also well defined [1, 2]. For example, in forensic toxicology, the primary question of an examination is to determine the presence and/or level of drugs or chemicals in the body of an individual. Forensics ballistics primarily answers questions about the association of a bullet with a firearm or about the trajectory followed by a bullet [3].

From the perspective of digital forensics, summarizing the questions answered by the field is almost impossible because the field covers any possible evidence that exists in the form of bits and bytes; no specific questions are defined for the field. Questions are often posed that apply to very specific circumstances and are sometimes not limited to the digital space. Often, the questions also deal with solving crimes, despite the National Academy of Sciences (NAS) warning about the tendency to use digital forensics to investigate crimes rather than examine evidence [4].

Digital forensics is, in practice, divided into subfields that are based on technologies from which evidence is being considered. A recent review of digital forensics models [5] described eight subdomains: database forensics, network forensics, memory forensics, small device and systems, computer forensics, mobile phone forensics, multimedia forensics, and software forensics; the review also explains how many of these can be further subdivided into other smaller subdomains. This focus on technology presents digital forensics as a field whose boundary spans the entire cyberspace because all technologies become subfields of digital forensics. Unfortunately, the nature of questions that can arise within cyberspace is dependent on countless factors, conditions, and technologies (both existing and evolving). The number of questions that can arise within cyberspace is also unlimited for the same reasons. At the same time, coherent *examination* techniques do not exist for most digital forensic disciplines.

This paper first presents a case for narrowing the questions answered in digital forensics. We posit that rather than considering cyberspace or digital technologies as the scope of digital forensic discipline(s), it is more appropriate to start with questions about digital events or artifacts where answers are deemed to be useful in a legal context, and then build a subdiscipline around closely related questions (where techniques can be developed to answer such questions). This is similar to the way subdisciplines of physical forensic science developed. The need to associate an individual with a crime scene has evolved into several disciplines, such as DNA analysis, fingerprint examination, and bitemark examination (the current controversies about bitemark analysis are not important in the context this paper uses bitemark examination as an example). We also posit that physical forensic disciplines that may have a digital equivalent may be a good starting point for a better delineated digital forensic subdiscipline. Questioned document (or forensic document examination) is a good example; physical documents have largely been replaced by digital documents, and it is necessary to be able to answer the same questions about digital documents that were useful to answer about physical documents (to the extent possible). Questioned document examination primarily speaks to the authenticity of a document, where authenticity needs to be defined in context. In the case of documents that are, in some sense, official, the term authenticity has its usual meaning. In the case of, say, a ransom note, the meaning of authenticity may be less clear. When a document is attributed to a known forger, the process of associating (forged) the document with the forger may speak to the “authenticity” of the forgery in an almost perverse manner. The question now is to what extent questions that can be answered by (physical) forensic document examination can be answered in the digital domain. A related question is whether the digital domain poses new questions about documents that were unanswerable in the physical domain. This paper only briefly reflects on the extent to which a field that we will call *questioned digital documents* (QDD) or *forensic digital document examination* (FDDE) seems viable.

In the latter part of this paper, we focus on the examination of questioned documents that have been customized from a database and explore approaches for answering questions relating to their authenticity. Like the physical domain, we adopt the use of exemplars. However, unlike the physical domain, we consider the implications of elements such as the database, document template, and the software used to create a customized digital document. We formalize the process of recreating such a questioned document to generate an exemplar for comparison and describe the level of accuracy in terms of the feasibility and level of conclusiveness in the processes. Our experiments portray the feasibility of the proposed method for FDDE. Although our experiments deal with the examination of digital documents customized from a database, the underlying approach of finding equivalent aspects from both physical and digital perspectives, and starting examinations with questions that are useful in a legal context transcends the field of questioned digital documents.

## 2 RELATED WORKS

**FIGURE 1:**
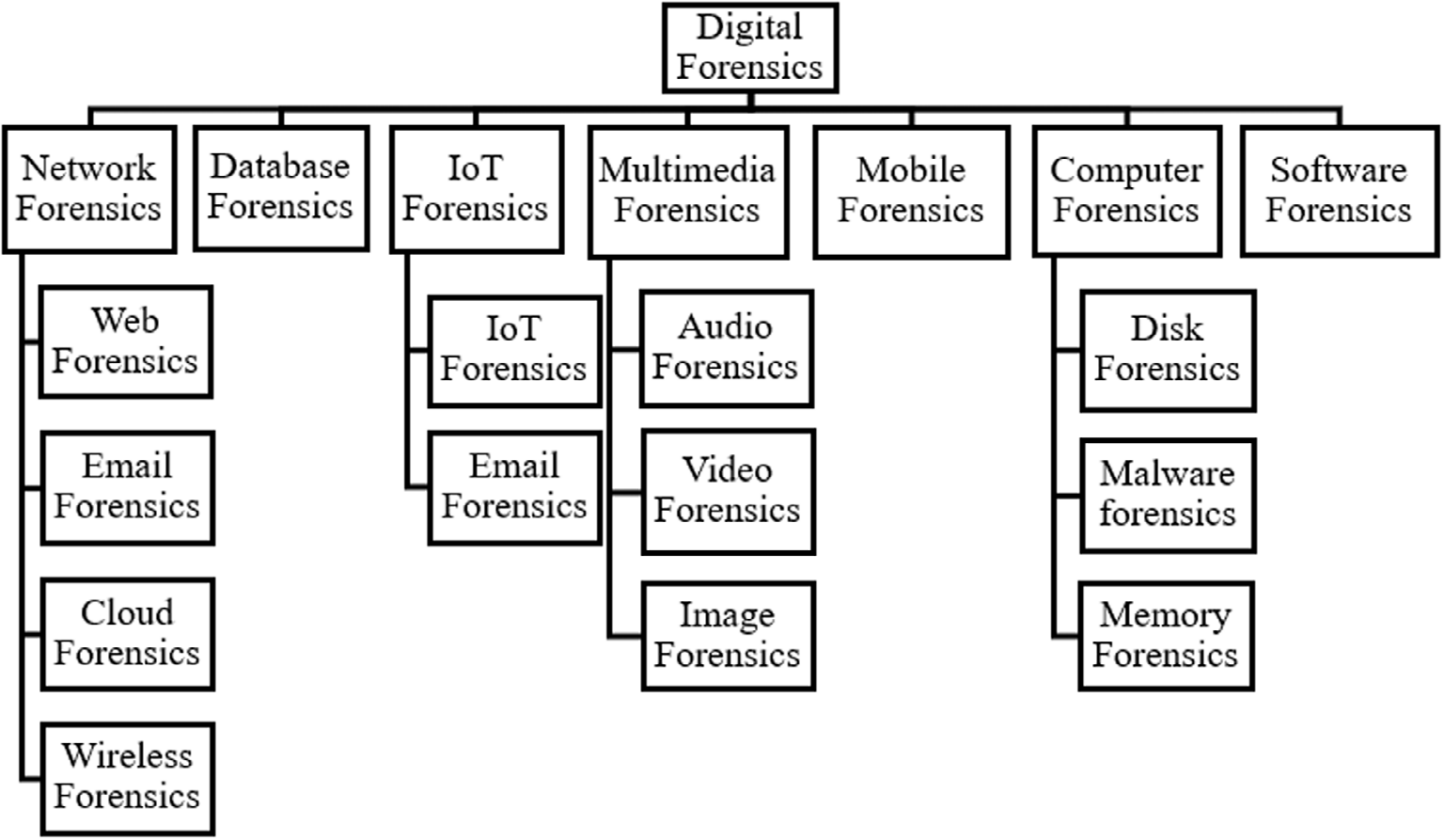
Subdomains of digital forensics.

Much of the earlier research in digital forensics was intended to facilitate the investigation of traditional computer crimes [5, 6]. According to a report published by the US National Academy of Sciences in 2009 [4], the goal of most digital forensic examinations was to find files with potential evidence and discover information about when and how the files came to exist on the computer. Today, digital forensics has a myriad of applications that stem from the advances in networked and distributed computing, wireless and mobile communications, portable electronic devices, the Internet of Things (IoT), and other technological developments. As the number of security incidents involving various digital subsystems continues to increase, many digital forensics models and techniques [5], as well as subdomains for addressing different aspects of digital examinations have evolved. Figure 1 depicts some of the existing domains of digital forensics.

In many subdomains of digital forensics, analysis techniques focus on a specific subspace of cyberspace; questions attempt to answer questions that arise within such a subspace. Network forensics, for example, involves the analysis of network‐related information. According to Palmer [7], it has the purpose of “uncovering facts related to the planned intent, or measured success of unauthorized activities meant to disrupt, corrupt, and or compromise system components as well as providing information to assist in response to or recovery from these activities.” This suggests that network forensics addresses the who, what, why, where, when, and how questions. This already deviates significantly from most branches of physical forensics. A DNA examination, for example, does not speak to “planned intent,” “measured success,” or “recovery from” unauthorized activities. Neither does toxicology, nor does ballistics. A medical postmortem may speak to the *how* and *when* questions. Where necessary, the *who* question is typically addressed by another forensic discipline (such as DNA analysis or forensic dentistry). Similarly, the *where* question may, when necessary be referred to entomology or other disciplines that speak to traces from the environment left on the body. In rare cases, the pathologist can speak to the *why* question, such as where a murder was exceptionally brutal, but human intention is rather the field of expertise of forensic psychology.

Other digital forensic subfields often also attempt to answer the gamut of the who, what, why, where, when, and how questions. Although the common approach to addressing these questions has been suggested to be through traceback techniques [8] (in network forensics for example), the number of questions that can be asked is unbounded and depends on the individual scenario being investigated [9, 10]. Coupled with the existing challenge of understanding what information is to be collected in an investigation [7], this absence of common clearly defined questions answered by the field limits the ability to provide a common understanding of the accuracy of analysis approaches. Mobile forensics, as another example, focuses on recovering evidence from mobile devices. As seen in existing work [5], the goal of analysis in this subdomain is often to solve a specific problem or retrieve specific data. This can be seen in some research works [11, 12] that are only applicable in certain situations or for specific types of mobile devices.

Similarly, many other subdomains of digital forensics also address investigations relating to a specific aspect of computing and a wide variety of questions depending on each case being investigated. While this has been instrumental in advancing the digital forensics field, the wide range of questions suggested by the discipline makes it hard to have a clear definition of questions that it addresses or to define criteria for the accuracy or correctness of analysis techniques in different subdomains. In the following section, we make a case for considering an alternative approach to digital forensics that aims to address this challenge.

## 3 AN ALTERNATIVE APPROACH

As discussed earlier, many subdomains of digital forensics do not have a well‐defined set of questions that are answered by the field but instead address each investigation on a case‐by‐case basis. This may be attributed to the fact that the digital forensics discipline is deemed to be applied in the digital space or cyberspace, which effectively implies that we need to investigate matters within the “space.” The challenge with approaching digital forensics from this perspective is that the definition of digital space or cyberspace is inadvertently too wide to define a concise set of questions or derive specific methods that apply across several possible instances of forensic examinations in each subdomain or across the different subdomains of digital forensics.

**FIGURE 2:**
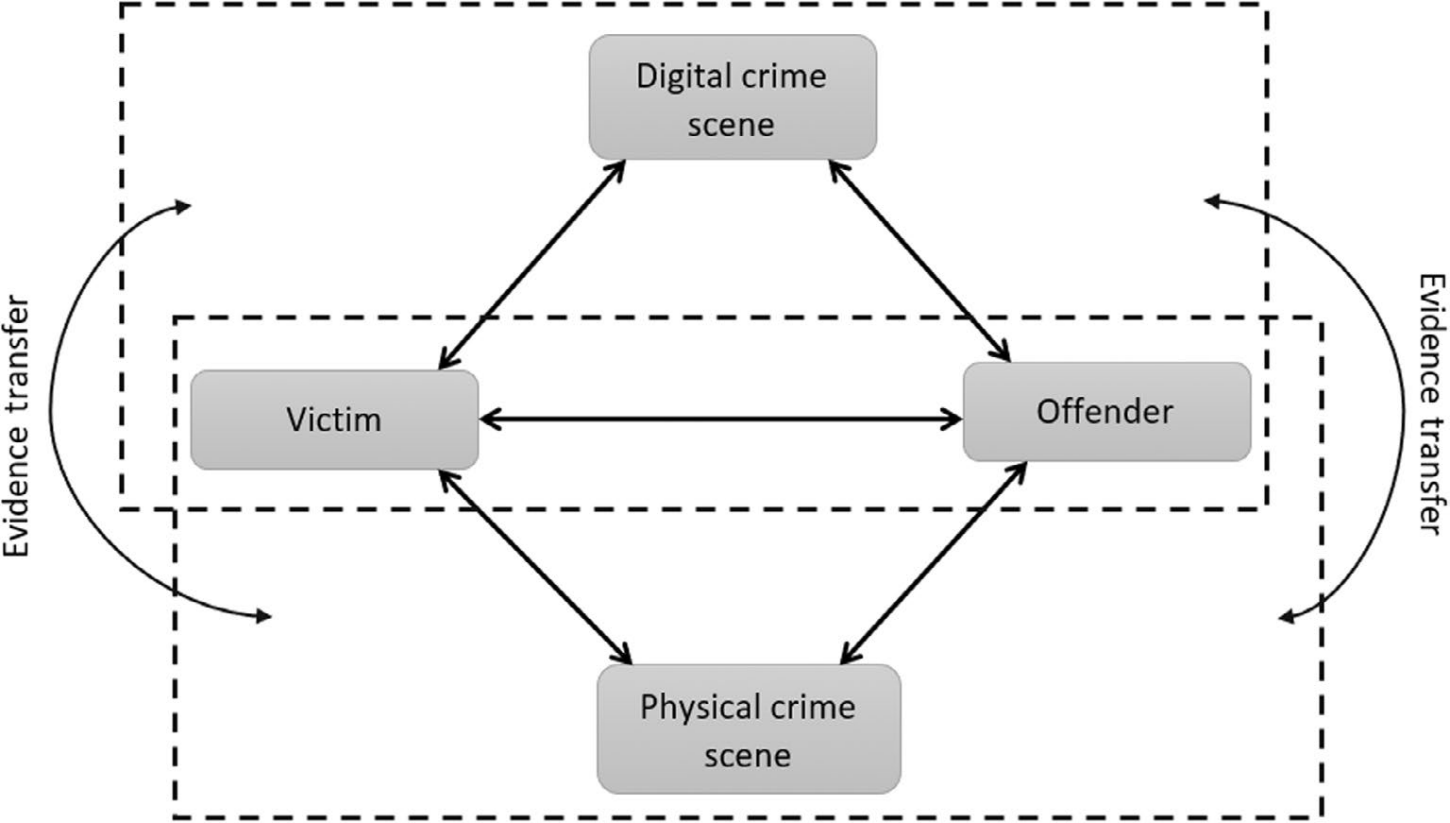
Physical and digital crime scene interaction.

This is in contrast to other forensic science disciplines such as toxicology, ballistics, and questioned documents where there is a clear understanding of the questions addressed by each discipline. One common attribute of these fields is that the source of potential evidence is somewhat bounded and may be envisaged from a high‐level description of the case being investigated. Conversely, identifying potential sources of evidence in digital forensics requires a detailed understanding of the interaction between the victim, offender, physical crime scene, and the digital crime scene as shown in Figure 2 [13]. Within the digital crime scene itself, a further detailed understanding of how several possible subsystems may interact with one another is also crucial.

Rather than focusing on several subsystems, we propose that an alternative approach to addressing digital forensics research and examinations is to consider the digital aspect of other disciplines of forensic science where the questions addressed are clear and concise, and where issues about the accuracy and reliability of claims and approaches have a better basis of measurement or evaluation. Several possibilities exist when we consider digital evidence in terms of the application domain rather than the environment or potential location of the evidence. For example, digital ballistics has received some research attention [14]; also, by building on Locard's principle, notions from physical toolmark analysis can be applied to digital toolmark analysis [15].

We note that this does not necessarily imply that we need to develop completely new approaches or techniques for digital investigations. In some cases, we may have already indirectly explored some of the interactions between the physical and digital dimensions. However, this alternative approach to digital forensics provides an opportunity to formulate precise questions and evaluate the accuracy of claims made in investigations, resulting in a more unified analysis approach similar to other disciplines of forensic science. Although it will be necessary to flesh out the digital aspects of some disciplines, others may be more comprehensible from a digital perspective. For example, the notion of questioned digital documents can be considered as an alternative approach to integrating digital forensics and the questioned documents discipline. Questioned documents is a well‐developed discipline and addressing the examination of questioned digital documents from the perspective of questioned documents presents insights for integration as we show in the following section.

## 4 QDE AND THE DIGITAL ASPECTS

In this section, we explore some of the perspectives that can be considered in the examination of questioned digital documents: First by describing the key goals and classes of the questioned documents discipline and then analyzing these from a digital perspective. We highlight some relevant existing works and provide an understanding of different aspects that can be considered for such integration.

In broad terms, the field of questioned documents provides answers to questions about the authenticity and origin of a document. A forensic document examination often involves the comparison of handwriting and signatures, analysis of alterations and obliteration of documents, counterfeiting, photocopy manipulation, rubber stamp impressions, paper, inks, and other types of analysis to determine details about a document [16] and is used to support claims in an investigation. Whereas there exists a wide variety of questions that could be addressed in digital forensics, the questioned document field is one where the questions that can be answered are clearly defined. From the perspective of documents, following an alternative approach to digital forensic examinations involves exploring some of the key aspects of questioned documents and considering possible digital counterparts of these aspects as we show below.

### 4.1 Document origin

A common objective of questioned document examination is to ascertain the source or origin of a document. This may involve identifying the type of typewriter used or may involve questions about the material used in the production of the document. Although the traditional approach to addressing this problem often involves chemical, ink, and physical analysis [17, 18, 19, 20, 21, 22, 23], recent studies [24, 25, 26] based on the analysis of physical features such as local texture patterns, microscopic structure, and texture information with the use of deep learning systems have been proposed. In digital terms, identifying the origin of a digital document may imply the identification of the tool that was used to prepare or edit the document. Because tools that create or modify an artifact would leave marks on the artifact, this inherently becomes a question of analyzing tool marks left on a digital document to identify the tool.

Built upon a key premise that tools have and transmit distinct features that can be traced back to a particular tool, the concept of tool mark analysis [27] can be applied to the examination of digital documents to identify both class and individual characteristics that can be traced back (through comparison) to a particular tool or group of tools. From a digital document perspective, tool mark analysis may involve answering questions about whether a document was prepared (or has been modified) using specific software, identifying the class of software that may have created a document, or the specific version of the software that was used to create a document.

### 4.2 Document age and alterations

**FIGURE 3:**
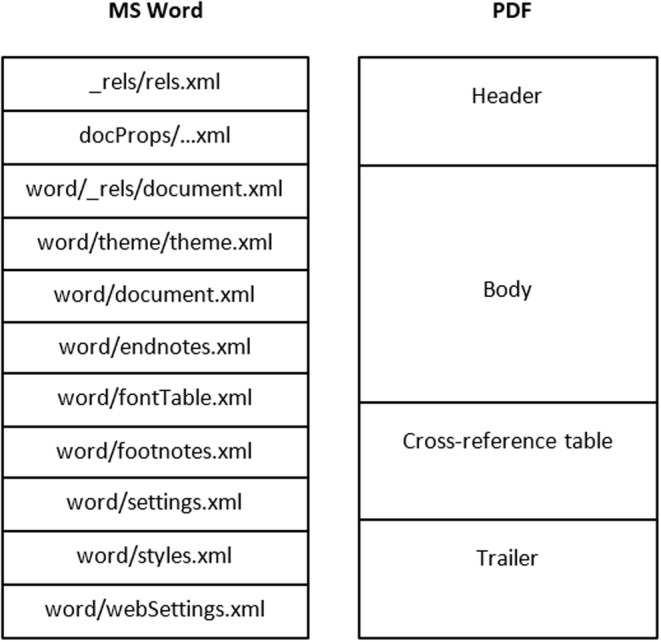
Comparison of MS Word and PDF file structures.

Determining the age or date of a document has been one of the most important challenges for questioned document examination and can involve efforts to identify a document's date through the writing or printing sequences on it [28]. From the perspective of a digital document, metadata information in a document may be useful for identifying the creation or modification date of a document. Metadata analysis has been applied to subdomains such as video [29], image, and database forensics. However, the application of metadata analysis is subject to the reliability of such metadata. While this represents a challenge depending on its use, it also presents opportunities to identify alterations made to documents. Although metadata can be modified, the process of modification is not often an easy task unless completed by someone with some level of expertise and in many cases, such modifications may lead to inconsistencies in the metadata or changes in the expected behavior which can point to evidence of metadata tampering [30]. Similar to how ink and paper can be characterized in QDE, the metadata allows the characteristics of the tool used to create or modify a document to be established. A comparison of the expected composition or structure of the metadata from a purported tool with those found on a document can be useful for knowing whether a document has undergone some fraudulent alterations. Figure 3 shows an example of the expected composition and/or structure in MS Word and PDF documents. In our experiments section, we show how some of the internal structures of MS Word can be used for analysis. A recent work by Olivier [31] addresses the determination of the age of questioned digital documents.

### 4.3 Questioned signature

When addressing the authenticity of physical documents, the analysis often involves determining the authenticity of the signature on the document or answering questions about the handwriting on it [32]. This often involves studying an individual's writing style, the characteristics of their signature and its variations, and a comparison of known normal writings to the questioned one. Recent studies have also explored similar approaches and feature extraction techniques for analyzing physical documents containing online or electronically written signatures [33, 34].

The digital domain has an equivalent notion of digital signatures. However, authenticating digitally signed documents is a simple computational process, and there is a general acceptance that a digitally signed document is not modified after it has been signed because the signature depends on the private key of the signer and the document content. If we consider similar questions as in the physical domain, we may want to determine if the digital signature on a digitally signed document can be forged or if such a signature proves that the document was indeed signed by the person who allegedly signed it. Addressing the question of equivalence digitally implies asking if a document has been signed by the owner of a key. This is clearly a problem of non‐repudiation in computer security and has the broadly accepted solution of using digital signatures. As such, questioning the acceptability of digital signatures may be an issue that is better ignored.

Beyond these initial questions, however, other problems can emerge when dealing with documents that are digitally signed. One such problem that was investigated by Olivier [35] considers the fact that documents are often questioned a long time after they have been signed, for example, an old contract, a last will, or an old email. Cryptographic techniques tend to become weaker over time due to the availability of more computational power which may not have existed when the technique was first created. This would imply that a document signed, two decades ago, for example, may no longer be considered authentic because the signature cannot be trusted or the key has expired. Although approaches such as increasing key length, and replacing the key or the algorithm [36, 37] used for encryption can be useful for addressing this challenge in aspects such as network communication, many of these are not applicable to QDDE since it is possible that a questioned document only exists on a disk and the original signer is no longer available or the key was never replaced. Thus, addressing the authenticity of digitally signed documents in these scenarios requires further research.

### 4.4 Other digital aspects of QDE

Although the QDE aspects discussed above have a relatively equivalent notion in the digital domain, other aspects emerge for which further research is still required in the digital perspective. One such aspect involves the use of notarized or certified documents and affidavits or declarations. Depending on the jurisdiction, individuals such as the commissioner of oaths or notary public are entrusted with the ability to certify copies of original documents and administer oaths. Such certified copies or sworn statements are then deemed authentic, and a copy of the document is kept in the archive for future reference. With the increasing use of digital documents and the need to authenticate documents where the original copy is only downloadable from an online source, some of the physical processes have been directly transferred to the digital domain due to the lack of well‐defined approaches for handling digital documents in this scenario. For example, a printed copy of an online document is sometimes considered as “original” even though techniques from QDE may not apply in this case.

Another aspect of digital documents that differs from their physical counterpart relates to a digital document's ability to execute or perform certain actions based on computer instructions or macro encoded into them. In contrast, physical documents do not have this ability, and thus such a scenario is not considered in the physical dimension. A particular area of interest here is where a document contains malware. Claiming that malware might be responsible for an action is often a common defense in investigations. The ability to prove the nature and capability of a document is thus important in taking legal action.

### 4.5 QDDE and other subdomains

Although some of the digital aspects considered in Section 4 still need more research, other aspects can be built on some of the solutions that have been proposed in subdomains such as multimedia forensics, file system analysis, and malware forensics. This section highlights some of the applicable existing works in these subdomains.

#### 4.5.1 Document types

In general, digital documents can be categorized into one of two formats, that is, text or binary. However, some binary files, for example, PDF files, can contain significant amounts of text data; thus we also consider a third category of documents, known as text‐rich files. Although all files consist of data stored as a series of bits, text file bits represent characters while binary file bits represent some custom data. Text files contain only textual data and use a standard simple format and as such can be read and edited by several programs or text editors. A binary file may include several types of data such as image, video, and audio in the same file. While the information can be interpreted by a supporting software, the data are mostly not readable in a text editor but the contained textual information may be readable.

From a forensics perspective, many of the existing approaches for digital document examination have focused on binary files, specifically multimedia documents, for example, images, video, and audio. The field of multimedia forensics addresses some of the aspects earlier described above for QDDE. The fields of file system forensics and malware analysis also address some of the aspects earlier described. Below, we highlight some of the research in these fields, not as an exhaustive description of the fields but to emphasize some applicable works toward the digital aspects of document examination earlier described.

#### 4.5.2 Multimedia forensics

Multimedia forensics has mostly been focused on identifying fraudulent alterations through passive techniques that aim to analyze the traces introduced during the acquisition, creation, or editing of multimedia documents, particularly, images. Over the years, approaches for copy‐move detection, splicing detection, compression detection and detection of retouching, lighting, and other inconsistencies have been proposed [38, 39]. While it may be possible to adapt some of these approaches to the authentication of other document types, there is still a need for such research. Other passive approaches [40] that address the determination of the origin of multimedia documents have been focused on identifying the acquisition device. However, research into the identification of tools, that is, software that has modified a document has not received a lot of attention. The ability to perform such an identification has the benefit of providing further evidence in attributing modifications made to a digital document.

The use of active techniques such as digital watermarking and digital signatures as defensive measures to protect documents and support the determination of their origin or legitimacy at a later time [38] has also been explored in multimedia forensics. The application of digital signatures for document authentication can be extended to any type of document but as earlier discussed, understanding how to address cases where the original signer is no longer available is an area that still needs to be considered. Many of the QDDE aspects earlier described will also benefit from the tremendous growth of machine learning‐based techniques which have been seen in multimedia forensics in recent years [41, 42]. However, as noted by Olivier [43], it is important to keep reservations about the use of machine learning in mind when dealing with evidence to ensure its reliability and freedom from bias that may be introduced through patterns in the dataset involved.

#### 4.5.3 Malware and file identification

Malware analysis involves the use of either static or dynamic analysis approaches to determine whether a file or document is malicious. Whereas static analysis relies on the ability to retrieve certain characteristics and features that provide some insight into intended malicious activities, dynamic analysis approaches involve the execution of the code to observe its actions [44, 45]. Several machine learning methods that automate the analysis have also been developed over the recent years [44, 45, 46, 47]. Although many of the existing analysis approaches focus on the analysis of portable executable files, the examination of digital documents may require analysis of non‐executable or non‐binary files which may potentially lead to malware through other vulnerabilities in their processing environment. Understanding such potential behavior and having the ability to provide evidence to support this is still an area that requires further research despite the increasing work in malware analysis and understanding of vulnerabilities such as buffer overflows.

Automated file type identification is another area of research that has addressed some aspects of digital document examination relating to document origin and has also seen advances in the application of machine learning. Much of the research in this area has also mostly focused on the analysis of binary files and involves methods that rely on the use of metadata such as file extensions and header or footer signatures, file fragments, and other binary information. However, research is still required to develop approaches for analyzing text‐based files or the textual component of text‐rich documents to support their automatic identification and the examination of digital documents of all types. In addition, studies that address the identification of relevant documents for investigation have focused on how data collected for an investigation can be classified or clustered based on their metadata, contents, or keywords [48, 49, 50] or other intrinsic properties [51, 52, 53]. While some of the approaches in these studies may be relevant for the examination of digital documents, many of the studies focus on specific document formats. We posit that further research into other aspects of digital document examination will lead to improved approaches for addressing documents of all different types and formats.

## 5 PROPOSED METHODS FOR QDD CUSTOMIZED FROM A DATABASE

Regardless of the digital forensics subdomain, the need to examine documents of different types forms an important part of many investigations because digital documents may originate from different sources. Although the examination approaches for documents from different sources will be related, investigative techniques existing in a specific domain may also be instrumental for the examination of documents in such domain. In the remainder of this paper, we describe examination approaches for questioned digital documents that are customized or personalized from a database. We incorporate some of the digital aspects of questioned documents as previously described and some of the existing methods for database forensics. This section also extends our previous work [54] which we presented at the American Academy of Forensic Science annual meeting in 2022.

### 5.1 Database‐customized documents

Database systems play a critical role in storing data across different functions and departments in many organizations. Stored data are often used to create a variety of documents that play a central role in daily transactions and may be shared with stakeholders both internally and externally. In many cases, documents such as sales proposals, non‐disclosure agreements, invoices, bills, and electronic cheques have a specified template that is populated with information from a database to generate personalized documents for each stakeholder. Although the use of a template to generate documents can be achieved in several ways including through web applications, reporting or publishing tools, document generation software, etc., one popular use case is through the mail merge feature in MS Word. Regardless of the application or database involved, customized documents generated through a template can be questioned similarly to other document types. However, unlike other questioned digital documents, customized documents can be examined based on the template and the database involved.

The field of questioned documents and other existing works relating to digital document examination focuses on comparing a questioned document with some exemplar or other version considered the authoritative version. In the case of documents customized from a database, the original document created via a template and populated with information from the database is considered the authoritative version of the document. However, because customized documents are typically generated on demand, an authoritative version other than the document being questioned may not exist. When such documents are questioned, the examiner is faced with the challenge of determining whether the document was derived from the template and whether the document contains similar data as stored in the database instance at the time the document was created. An important factor in the examination process is identifying whether information about the document's date of creation is known or can be determined. The examination process relies on the application of different approaches depending on the state and availability of the document template, the software used to create the document, and the database from which the document was customized. To provide a discussion of these methods, we formalize the concept of a customized document and their creation as follows.

### 5.2 Formal definition

A customized document *D* is a document that is created from a template document *T*, and a database content *C* using a software *S*. Given a specific time *i*, $T_{i}$, $C_{i}$, and $S_{i}$ represent the template at the time *i*, the database content at time *i*, and the software version at time *i*, respectively. The creation of a customized document *D* can be denoted by the expression:

(1) $$D=S_{i}\left(C_{j},T_{i}\right)$$

The examination process described in the following sections relies on the ability to authenticate a questioned document through a binary comparison with a recreated document. We define a recreated document (exemplar) $D^{\prime }$ using a similar expression as in Equation (1):

(2) $$\begin{array}{c} D^{\prime }=S_{j}\left(C_{k},T_{l}\right) \end{array}$$

where *j*, *k*, and *l* may either be equal or not and may differ from *i*. $S_{j}$ represents the version of the software (at time *j*) that is used to generate the recreated document, $C_{k}$ is the content (at time *k*) of the database used, and $T_{l}$ is the template (at time *l*) used to generate the recreated document.

Given a dated customized questioned document, we consider different scenarios that determine the analysis steps followed in authenticating the document. These scenarios are based on whether or not the software, template, and database content are available for a document to be recreated. Where the original software version, template, and database content used to create the questioned document are the same, it implies the $S_{i}=S_{j}$, $C_{i}=C_{k}$, and $T_{i}=T_{l}$. Although the availability of these elements leads to three key scenarios, a combination of two or more of these scenarios creates other situations that are worth exploring. Table 1 provides a summary of the possible combinations of scenarios (based on whether the original software, template, or database content used to create the questioned document is available), the feasibility of recreating the document, and the analysis approach that may be used in each case.

**TABLE 1:** Feasibility of document recreation and the recreation approach for dated documents.

Case	$S_{i}=S_{j}$	$T_{i}=T_{k}$	$C_{i}=C_{l}$	Document recreation
1	Yes	Yes	Yes	Feasible $D^{\prime }=D=S_{i}\left(C_{i},T_{i}\right)$
2	No	Yes	Yes	Feasible $D^{\prime }=S^{\prime }\left(C_{i},T_{i}\right)$ if $\exists S^{\prime }$
3	No	No	Yes	Partly feasible Cannot be automated
4	Yes	No	Yes	Partly feasible Cannot be automated
5	Yes	No	No	Not feasible
6	Yes	Yes	No	Partly feasible $D^{\prime }=S_{i}\left(C^{\prime },T_{i}\right)$ if $\exists \;C^{\prime }$
7	No	Yes	No	Partly feasible $D^{\prime }=S^{\prime }\left(C^{\prime },T_{i}\right)$ if $\exists \;C^{\prime },S^{\prime }$
8	No	No	No	Not feasible

Given an undated customized questioned document, the age of the document may first be determined. Olivier [31] described how the principle of inclusion may be used to determine the creation date window of a questioned digital document. With a reconstructed creation date or window *w* for an undated document, such that i⊆w, the same approach as detailed above can be used by treating the document as a dated document so that the document can be reconstructed as

$$D_{w}=S_{j}\left(C_{k},T_{l}\right)$$

where j⊆w, k⊆w, and l⊆w. This implies that the recreation process in this case would require that the template, within the determined document age, is available or there are exemplars from the time. It also requires that the database content during this time can be reconstructed if there have been changes to it. The aspect of reconstructing content is explained later.

**FIGURE 4:**
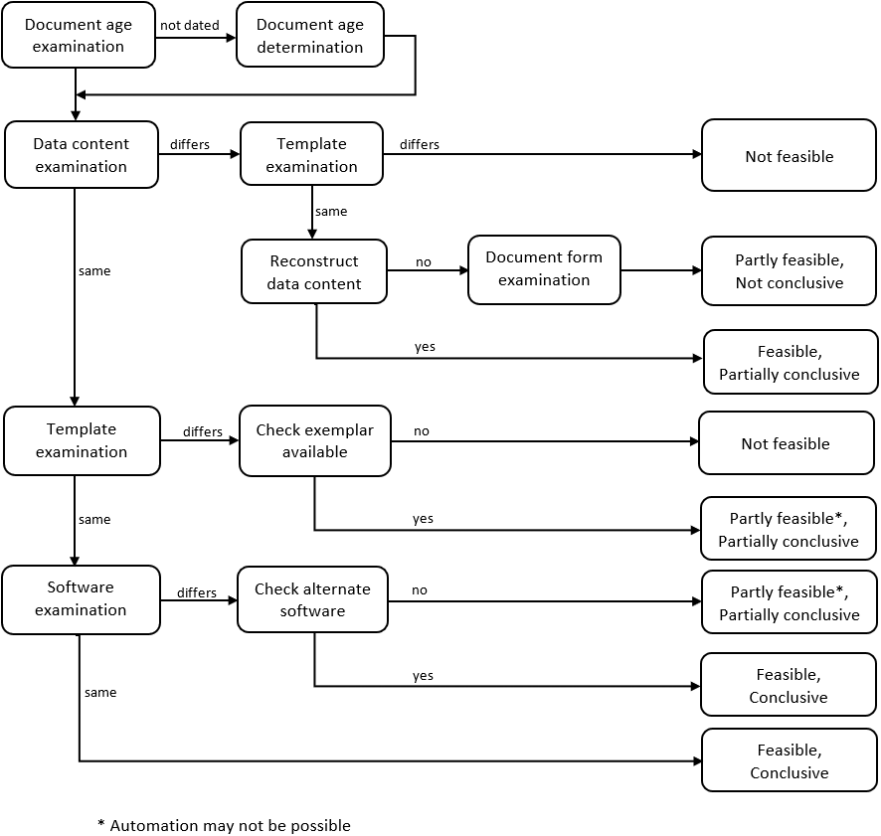
Document recreation and feasibility process flow.

Figure 4 presents a flowchart for the overall examination process for customized digital documents and outlines the steps involved, as well possible outcome of the document recreation process in the cases identified in Table 1. In the following sections, we discuss the process of examining the template, database content, and the software in relation to each of the cases above. Some relevant background for each of the cases and examination approach are also described.

## 6 DISCUSSION

Given the reconstruction definition in Equation (2), generating a recreated document and comparison with the questioned document is feasible and conclusive when the software, template, and database content are the same or an alternate version of the software $S^{\prime }$ that works similarly to $S_{i}$ is available. As noted in Section 8, research is still required to identify the software used to create a document in cases where the software that created a questioned document is not known. If the available software works differently relative to the original, the document recreation is still partially feasible, depending on an understanding of the difference between both software versions. The comparison is also partly conclusive and may involve some manual processes.

In cases where both the template and the database content are no longer available and cannot be reconstructed [55], the document recreation and comparison process becomes infeasible. All other scenarios rely on the ability to either reconstruct the database content or rely on the use of exemplars created from the same time frame as the questioned document. If the database content is available but the template is not, the recreation and comparison process is partly feasible and relies on the use of exemplars for the comparison. The examination is also partly conclusive. The comparison process in this case can be applied in a relatively simple way such as doing a comparison of the perpetual text in the exemplars to the questioned document. More advanced comparisons may depend on the internal structures and the type of the files being compared. Much work has been done on analyzing specific file formats, and this information can be applied to comparing two files of the same format. For example, in [44, 51, 56], the authors provide information about the structure of PDF, MS Word, and OOXML files which can be used to compare the structure and content of these files. They explain how the file structure can be useful in forensics investigations. This knowledge about the file structure provides a basis for comparison of a file with the questioned document, in terms of both the textual data and beyond it. As shown in our experiments in Section 7, when examining documents generated from an MS Word template or exemplar, the revision identifier associated with the files can be explored to identify similarities between a template and a questioned document that is purportedly generated from it, or between exemplars generated from the same template. The document recreation process in this case cannot be automated, but the comparison may still be automated. An alternative comparison method that can be used when the database content is available is to explore the dependencies of the document fields and those of the database content. Some interdependencies that can be used for this purpose include primary‐key/foreign‐key relationships, parameters (and their data type) implied by any functions or stored procedures in the database, and the types of data that any existing database views or temporary tables provide. For example, a field that infers the total telephone charge to a customer may be verified by querying for the records relating to this customer in the database. Also, if the schema in a relational database specifies a field as a foreign key in a table and the field is used in the template, queries that check that the foreign key does exist in the associated table with the primary key can be executed.

In cases where the database content is not available but the template is available, a reconstruction of the data can be used to facilitate the document recreation and comparison process. This approach is also relevant when an examination involves a large number of documents or files with large sizes. The ability to reconstruct the necessary data makes the document recreation possible and allows the recreation and comparison processes to be automated. In our earlier work [55, 57], we showed that data in a database can be reconstructed in situations where the goal is to recover specific information. We introduced the notion of relational algebra log, value blocks, and inverse relational algebra [55], and described different approaches that exploit the underlying mathematical construct of relational database systems. As noted in the earlier works work, the aim of applying inverse operators is to find the value of an attribute (for a tuple in a specific relation *R*) at a specific time of interest. Although the inverses may generate incomplete data, the data reconstruction approach can be applied for the recreation of documents for comparison because the problem aligns with the goal of reconstructing specific values existing in the database at an earlier time of interest. We note that, since our interest is to reconstruct data that form part of the questioned document, the need for a complete reconstruction of the entire database state or an entire tuple in the database is eliminated. Details of our data reconstruction process can be found in previous works [55, 58, 59, 60]. If the data of interest cannot be reconstructed, the document recreation process remains partly feasible by employing some of the approaches earlier described, as applicable. However, the comparison process is not conclusive because only the format or structure of the questioned document may be examined in this case, the data content cannot be examined.

## 7 EXPERIMENTS AND RESULTS

Our experiments were conducted using MS Word templates and customized documents generated through a database created using MS Access. The use of MS Word templates with many database management systems is popular. We note, however, that the underlying analysis approaches used in the experiments, as previously discussed, are not dependent on the application used as a different database management system or template file type could be used. We considered different versions of our template document, customized documents, and exemplars to examine the methods and scenarios earlier discussed.

**FIGURE 5:**
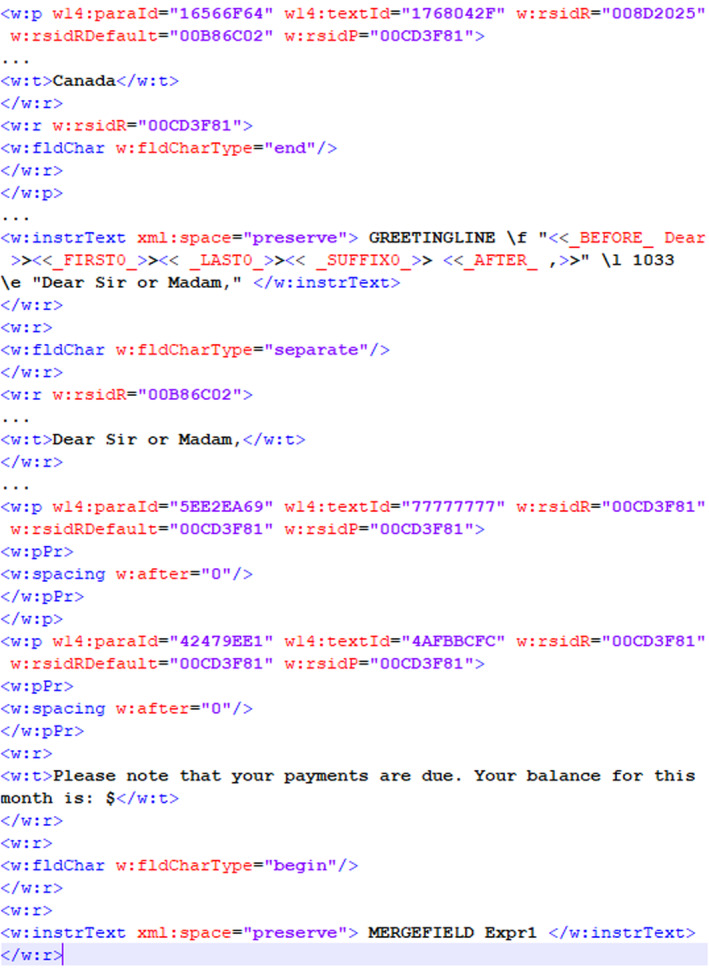
RSID in template document.

**FIGURE 6:**
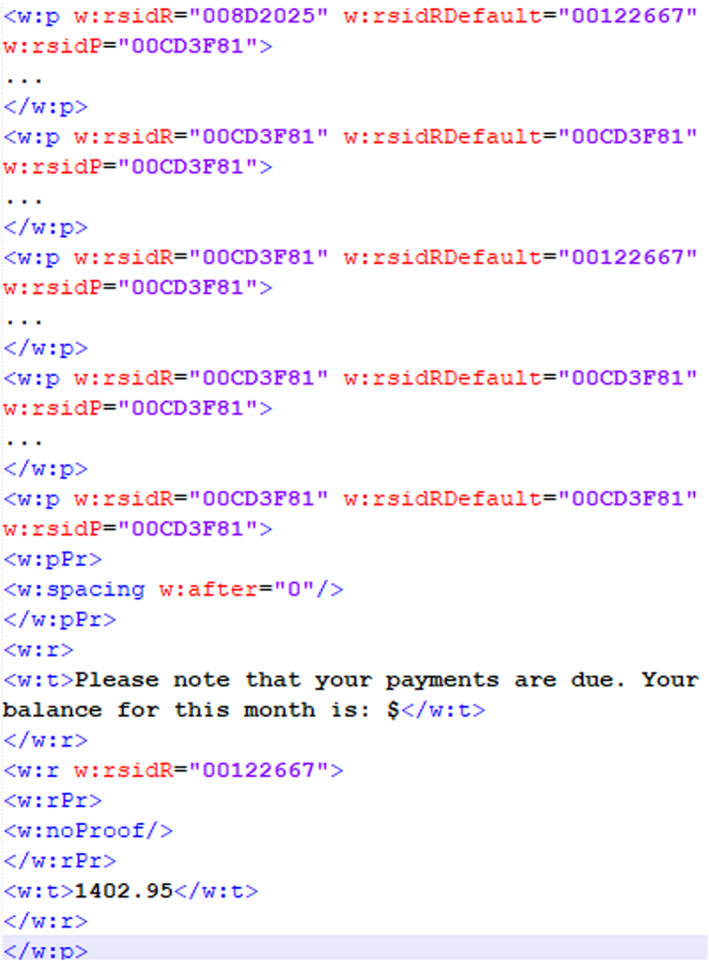
RSID in customized questioned document.

**FIGURE 7:**
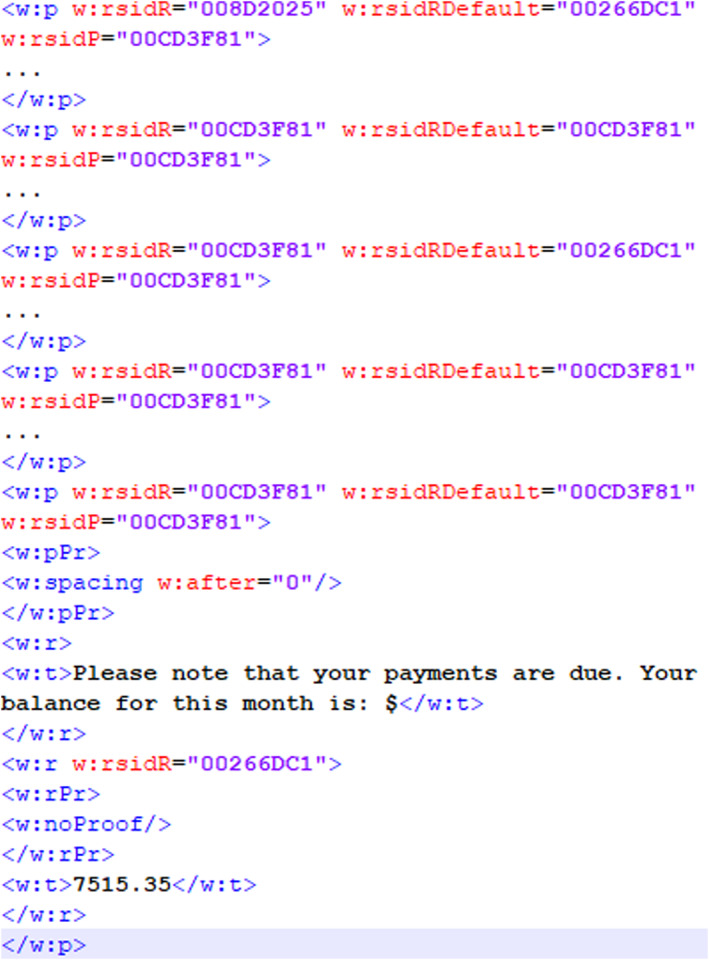
RSID in exemplar document from similar template.

In our experiment to explore the direct comparison of a questioned document with a template, we created an MS Word template that is used with a database to generate customized documents. We explored the use of the revision identifier (RSID) that exists in MS Word documents to determine whether a questioned document is based on a template document. A revision identifier is a 32‐bit value that is stored inside the document file whenever it is modified or saved [51]. Figures 5 and 6 show similar revision identifiers in both documents, for example, “008D2025” and “00CD3F81” occur in both the template and questioned document. The RSID “00CD3F81” occurs in every paragraph of both documents, signifying that every paragraph started from the same source template. The template document in Figure 5 also contains RSIDs and data that show the fields and or paragraphs that may be modified based on the data available in the database on which the template is used. Figure 6 shows additional RSIDs in the customized document together with some of the original RSID values in the template document based on the information that is added to the customized document. Although the RSID corresponding to the updated fields changes, Figure 7 shows that the original RSIDs are retained for all documents generated from the same template.

In situations where the software, template, and database content remain unchanged (Case 1), we successfully recreated an exemplar and performed a direct comparison with the customized document. In Case 2, where the software or the software version was not available, the recreation was completed using an earlier version of MS Word. An alternate software that provides some similar features to MS Word (e.g., LibreOffice Writer in this scenario) was also successfully used for the recreation process. In the case where only the original data content is available or unchanged (Case 3), the recreation of the entire document could not be completed or automated but the inferred information in the questioned document was determined from the data. For example, for our sample template (Figure 5), the inferred information or data of interest is the monthly balance due. This information is determined by executing a query to generate this information. This is feasible since the data of interest in the database have not been modified. However, the constant text in the questioned document cannot be verified since the template is no longer available. A similar outcome also occurs where both the software and data contents exist but the template does not (i.e., Case 4). Although the availability of the software or its alternative enables the automation of the recreation process, in situations where both the template and the data contents are no longer available (i.e., Cases 5 and 8), we were unable to recreate an exemplar, as depicted in Table 1.

In the last two scenarios (Cases 6 and 7) where the template is available but the content has changed, we employed database reconstruction to facilitate the document recreation process. This had partial feasibility depending on the output of the data reconstruction. Based on the template document (Figure 5), the inferred field in the database for a customer's total bill amount, for example, is the “Expr1” field. When the corresponding value at the creation time of the questioned document (i.e., the time of interest) has been modified in the database, the value may be determined by reconstructing the values for the fields that make up the expression. In this case, the “Expr1” is given by “Sum([unitprice]*[quantity]),” implying the total amount spent by a customer on some purchases and we can regenerate this value for comparison. Without the template or where the template is considered different, information about the inferred field cannot be determined, making the examination infeasible. As shown in our earlier work [55, 60], inverse operators of the relational algebra can be applied to operations that have modified these fields to determine the values at the time of interest. Table 2 summarizes the inverse operators described in our earlier work [58] (where, *R*, *S*, *T* are relations and *A*, *B*, *C* are attributes). For example, in a scenario where the database log contained a query that inserts all purchase records of the customer of interest into a new table *T* together with some other records, we used the inverse union operator $\cup^{-1}\left(T\right)$ to reconstruct only the records belonging to the customer of interest and recalculated the value of “Expr1.” Given that the table containing the [unitprice] and [quantity] fields has a foreign key with associated records in a table *S*, we could also extract more information by querying *S* with the foreign key as a selection criterion.

**TABLE 2:** Inverse operators of the relational algebra.

Operators	Query	Inverse operators
Rename $\left(\rho \right)$	$R\leftarrow \rho_{A_{i}=B_{j}}\left(R\right)$	$\rho^{-1}\left(R\right)=\rho_{B_{j}=A_{i}}\left(R\right)$
Cartesian product $\left(\times \right)$	$T\leftarrow R\left(A\right)\times S\left(B\right)$	$\times^{-1}\left(T\right)=\left(R,S\right)$ where $R=\pi_{A}\left(T\right)$ and $S=\pi_{B}\left(T\right)$
Union $\left(\cup \right)$	T←R∪S	$\cup^{-1}\left(T\right)=\left(R^{*},S\right)$ where $R^{*}=T-S$, if S is known and vice versa
Intersection $\left(\cap \right)$	T←R∩S	$\cap^{-1}\left(T\right)=\left(R^{*},S^{*}\right)$ where $R^{*}=S^{*}=T$
Difference (−)	T←R−S	${-}^{-1}\left(T\right)=R^{*}=T$ If R is known, $S^{*}=R-T$
Division $\left(/\right)$	T←R/S	${/}^{-1}\left(T\right)=\left(R^{*},S^{*}\right)$ where $R^{*}=\mathrm{RM}$, RM is the remainder of the division
Join $\left(⋈\right)$	$T\leftarrow R\left(A\right){⋈}_{p\left(A,B\right)}S\left(B\right)$ $T\leftarrow R{⋈}_{p\left(A,B\right)}S$	${⋈}^{-1}\left(T\right)=\left(R^{*},S^{*}\right)$ where $R^{*}=\pi_{A}\left(T\right)$ and $S^{*}=\pi_{B}\left(T\right)$
Projection $\left(\pi \right)$	$T\leftarrow \pi_{A_{1},A_{2},A_{3}}\left(R\right)$ $T\leftarrow R\left[A_{1},A_{2},A_{3}\right]$	$\pi^{-1}\left(T\right)=S^{*}=T$
Selection $\left(\sigma \right)$	$T\leftarrow \sigma_{p\left(A\right)}\left(R\right)$ $T\leftarrow R\left[p\left(A\right)\right]$	$\sigma_{p\left(A\right)}^{-1}\left(T\right)=S^{*}=T$

In general, if a template or questioned document was created with backward‐compatible software, a later version of the software can be used in the document recreation process. Because MS Word is generally backward‐compatible, we used an alternative later version to test the feasibility of recreating a document given that the template and data exist but the software is not. Despite that some templates were processed in the compatibility mode, the document recreation was completed successfully with conclusive results. Although backward compatibility is often supported in many applications, we note that the document age plays an important role in choosing an alternate software or software version.

## 8 CONCLUSION AND FUTURE WORKS

While we do not invalidate the current approaches to digital forensics research, which focus on several subdomains, we show in this paper that an alternative approach aimed at limiting the number and variability of questions and providing a way of discussing the reliability of techniques used in digital forensics is worth exploring, particularly in questioned digital document examination.

This paper discusses how an understanding of a relevant physical dimension or discipline may be useful in narrowing the questions that may be asked in the digital space and facilitating the discussions of accuracy and reliability. We showed an application of this approach to the examination of questioned digital documents by drawing from the questioned document field and outlining different digital aspects that may be encountered in questioned digital document examination (QDDE). The paper focuses on the examination of digital documents that are customized from a database to determine alterations to the questioned document. The detailed examination process shows how exemplars can be created and used for comparison to determine changes in a questioned document. A formal definition of the document recreation process and an analysis of their feasibility and level of accuracy is presented. The experiments conducted show the feasibility of this approach when dealing with documents customized from a database.

We note that the interaction and similarities between a physical and digital domain or discipline may require an in‐depth study of both dimensions. Although we fleshed out many of the digital aspects that may be considered for QDDE, more research is still required in exploring other digital aspects described in Section 4. An example that is relevant to the examination process described in this paper pertains to the determination of the tool that created a document. If the software that created a questioned document is not known, it may be necessary to determine this to find an alternative version of the software that works similarly. While many existing works in document analysis focus on file type identification, the identification of the software tool that created a document has not received enough attention and will be addressed in our future work. The exploration of other digital aspects is also necessary.

## FUNDING INFORMATION

This work was supported by the University of Winnipeg (Grant ID: 16792, 16673, 19685).

## CONFLICT OF INTEREST STATEMENT

The authors declare no conflicts of interest.
